# Protocol for geolocating rural villages of women in Liberia utilizing a maternity waiting home

**DOI:** 10.1186/s13104-019-4224-1

**Published:** 2019-04-02

**Authors:** K. H. James, J. E. Perosky, K. McLean, A. Nyanplu, C. A. Moyer, J. R. Lori

**Affiliations:** 1 Department of Learning Health Sciences, University of Michigan, Ann Arbor, USA; 2 University of Michigan School of Nursing, 400 N. Ingalls, Room 3237, Ann Arbor, USA; 3 Africare Liberia, Monrovia, Liberia; 4 Global Reach, Departments of Learning Health Sciences and Obstetrics and Gynecology, University of Michigan Medical School, Ann Arbor, USA

**Keywords:** Geographic information system, Maternity waiting homes, Liberia

## Abstract

**Objective:**

Geospatial data are used by health systems and researchers to understand disease burdens, trace outbreaks, and allocate resources, however, there are few well-documented protocols for collecting and analyzing geographic information systems data in rural areas of low- and middle-income countries. Even with the proliferation of spatial technologies such as Open Street Map and Google Maps, basic geographic data—such as village locations—are not widely available in many countries in sub-Saharan Africa. The purpose of this paper is to report a step-wise protocol, using geographic information system techniques and tools, developed to collect and analyze the type of spatial data necessary to calculate the distance between rural villages and maternity waiting homes located near rural primary healthcare facilities in Bong County, Liberia.

**Results:**

Using a step-wise approach incorporating local healthcare provider knowledge, intensive field work, and spatial technologies such as Open Street Map and Google Maps for village geospatial data collection and verification, we identified village locations of 93.7% of the women who accessed the five maternity waiting homes in our study from 2012 to 2016.

## Introduction

Liberia has some of the worst maternal health statistics, ranking 7th highest for maternal mortality in the world [1]. The country’s social and health structures were devastated during 14 years of civil wars and more recently, Liberia has been severely impacted by the Ebola virus disease outbreak. A notable 54% of births in rural areas occur outside the healthcare system without a skilled birth attendant [2], and nationwide 40% of women identify distance to a healthcare facility as a problem [2].

Maternity waiting homes (MWHs), small compounds built adjacent to healthcare facilities where women can stay prior to giving birth, have been proposed as an intervention to address the delay in reaching a healthcare facility [3]. Maternity waiting homes are seen as an important mechanism to reduce the impact of distance on women’s likelihood to deliver in a facility [4–6]. Yet data on how far women travel to stay at a MWH are scarce.

Previous research evaluated the impact of five MWHs positioned near primary healthcare facilities throughout rural communities of Bong County, Liberia. Results were positive, indicating a reduction in both maternal and perinatal mortality in clinics with an associated MWH [5]. However, the geographic distribution of the women utilizing the MWHs and the distances they traveled to reach a MWH and the adjacent primary healthcare facility for delivery has not yet been reported. This protocol describes a step-wise approach using geographic information systems (GIS) techniques and tools developed to collect and analyze the type of spatial data necessary to calculate the distance women traveled to utilize a MWH in Bong County, Liberia.

## Main text

### Methods

Geographic information systems are comprised of hardware and software specifically designed to allow users to input, store, analyze, and display geographic data [7]. GIS is commonly used to incorporate spatial analysis into public health practice and has been used in an array of global health applications including the management of tuberculosis [8–11], HIV [12, 13], and malaria [14–17]. Spatial analyses utilize data from diverse sources including satellite imagery, road networks, terrain elevation models, and existing global positioning system (GPS) information such as the locations of healthcare facilities or hospital catchment areas [7]. However, in many low- and middle-income countries (LMICs), basic GPS information such as the location of villages surrounding a health facility are not readily available.

This study took place in Bong County, Liberia, which has an estimated population of 333,481 [2]. Data on MWH stays between 2012 and 2016 at five original MWHs from a parent study serve as the study sites to investigate how far women who utilized a MWH traveled for facility delivery.

#### Data collection

Each MWH maintained a handwritten logbook of village names for pregnant women admitted to the MWHs from 2012 to 2016. Village names were collected from each logbook by members of the research team and entered into an electronic database. De-identified data such as mother’s home village, admission/departure date, and number of people accompanying the mother were recorded. The logbooks contained records for 2454 pregnant women, associated with 341 different villages names across the five MWH catchment areas. Using these data, our team created lists of mothers’ home villages for each MWH catchment area.

While the MWH logbooks provided initial lists of mothers’ villages, these data required extensive cleaning, organization, and processing. A step-wise approach was used in the field to ensure that village names and locations were correctly identified and collected.

#### Validity of GIS data sources

Three sources were used for generation of GPS data coordinates: collection of GPS coordinates in the field, Open Street Map (OSM), and Google Maps. Previous studies using GIS analysis in Africa have found Google Maps and OSM data to be valid, if not always entirely complete [18]. To ensure validity of the OSM and Google Maps data used in this study, a convenience sample of 49 village GPS points were collected in the field using hand-held tablets (Nexus 7). These 49 villages consisted of a list of the largest villages in their catchment areas. Using this field GPS data, the OSM and Google Maps data sets were determined to be accurate. Accuracy was calculated as percent error for latitude and longitude with 0.009 ± 0.01% and 0.004 ± 0.005% respectively for OSM and 0.037 ± 0.04% and 0.039 ± 0.04% respectively for Google Maps.

Following the initial identification and collection of village locations, GPS coordinates were assigned to the mothers’ villages identified in the MWH logbook if the spelling of the village name was an exact match. The remaining unmatched mothers’ villages consisted of apparent alternative spellings, misspellings of the villages whose GPS data had already been collected, or new additional villages whose names were not provided to the research team during the initial data collection process. For instance, in Liberia, ‘ta’ or ‘tu’ are often used interchangeably with ‘town.’ Therefore, apparent alternative spellings of ‘Charlie Town’ would include ‘Charlie-ta’ or ‘Charlie-tu.’ In other cases, there were obvious misspellings, such as a village listed as ‘Gartimon’ instead of ‘Garlimon.’

Following initial matching between logbook data and GPS data collected in the field, team members returned to the field and verified the entire list of villages from each MWH logbook with a group of 4–5 healthcare workers from the respective healthcare facility. Comparing the mother’s village list to an official list provided by the county health team helped determine whether unmatched villages were alternative spellings or if they were new unique villages. For example, healthcare workers were able to determine that ‘Dokai Ta’ and ‘Do Ki Ta’ were names used to refer to the same village, while ‘Loma-ta’ and ‘Loama-ta’ were actually two separate villages. Unique villages not in the original master village list were then added.

The logbook data (representing the 2454 women who delivered at the MWHs and the villages from where they came) were cleaned based on feedback received in the field, including modifying incorrect and alternative spellings to the accurate spelling of a specific village name. Next, misspelled villages were assigned the GPS coordinates of the correctly spelled village name. For example, ‘Gartimon’ was modified to the correct spelling of ‘Garlimon’ and was then assigned Garlimon’s GPS coordinates.

Following data cleaning and initial GPS assignment, OSM and Google Maps were used to identify the GPS coordinates for villages not captured in the field [19, 20]. Notably, the availability of publicly available GPS data in Liberia is relatively high compared to other African nations due to the extensive GPS mapping that took place during the 2014 Ebola Crisis [21]. Using the new, correctly-spelled village names, OSM and Google Maps were queried to identify missing villages’ locations. GPS coordinates from OSM or Google Maps were assigned to a village if the spelling of the village was an exact match and if the village was located in the appropriate catchment area.

Then, logbook entries were searched for variations in spelling for the remaining unmatched villages. For example, when searching for the village location for ‘Whennta,’ variations in the logbook included ‘Whenn-Ta’ and ‘Whenn Town.’

**Fig. 1:**
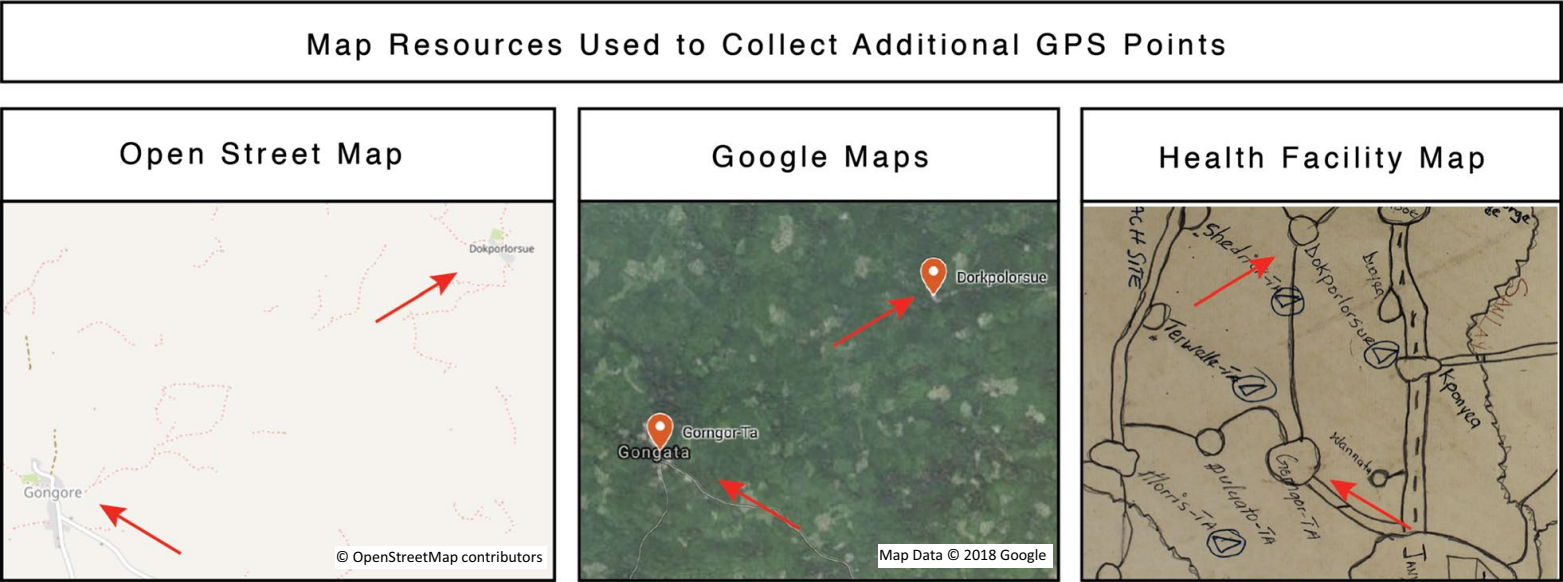
Map resources were used to collect and verify additional GPS points. Identified GPS coordinates were cross-referenced using Open Street Map, Google Maps, and hand-drawn health facility maps. The relative location of ‘Gorngor-Ta’ and ‘Dorkpolorsue’ are displayed as they appear in each resource. All map resources were used with permission

To find GPS coordinates for the remaining unmatched villages, hand-drawn maps created by the healthcare workers from each catchment area were cross-referenced with OSM and Google Maps. These hand-drawn maps, which are used to organize outreach activities, display the relative location of villages in proximity to each other. For example, the GPS coordinates for the village ‘Gorgor-Ta’ were identified in OSM listed under the name ‘Gongore.’ To verify that these coordinates were correct, the team referenced the hand-drawn map from the facility, which showed that ‘Gorgor-Ta’ is located southwest of ‘Dokpolorue.’ Both OSM and Google Maps confirmed this approximate location, confirming the GPS coordinates for ‘Gorgor-Ta’ (Fig. 1).

### Results

**Fig. 2:**
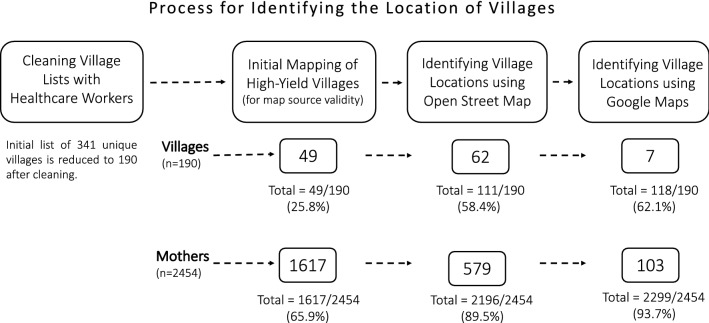
Process for identifying the location of villages

Following this step-wise approach, GPS coordinates were collected for the village locations of 93.7% of the women who accessed the five MWHs in our study from 2012 to 2016 (Fig. 2).

After reviewing the village lists with healthcare workers to identify potential alternative spellings of village names, the number of unique villages was reduced from 341 to 190. Based on identical spelling, initial matching between catchment villages and villages listed in the MWH logbook resulted in the GPS assignment of 49 villages. The 49 villages matched during this phase were home to 1617 of 2454 women using the MWHs for an initial match rate of 65.9%.

Next, OSM was used to identify an additional 62 villages, bringing the total number of matched villages to 58.4% and yielding another 579 mothers with an identified home village. The final round of GPS assignment using Google Maps yielded an additional 7 villages to bring the total number of matched villages to 118 out of 190 villages (62.1%). The additional 7 villages were home to 103 women (4.2%), bringing the total to 2299/2454 women with identified home villages (93.7%). The 93.7% of women with their home village identified came from 62.1% of the villages in the dataset. In consultation with the Bong County Health Team, none of the missing villages are documented villages in Bong County. Thus, it is assumed that these villages are outside of Bong County or are unofficial communities.

### Discussion

Public health practitioners have used geospatial data to understand disease burdens, design interventions, and allocate resources for centuries, however, there are few well-documented protocols for collecting and analyzing GIS data in rural areas of LMICs. Even with the proliferation of spatial technologies such as OSM and Google Maps, basic geographic data are widely unavailable in many countries in sub-Saharan Africa. This study reports on a step-wise approach that incorporated local knowledge, hand-drawn maps, field GPS collection, and digital technologies to ensure high-quality, accurate location data that will allow for higher order spatial analysis to determine how far women are traveling to utilize MWHs in Liberia.

### Conclusion

GPS data collection is a labor intensive and often expensive process, making it difficult for LMICs to maintain updated geospatial information and resources. Many of the challenges we faced in data collection and verification are not unique to Liberia and can be generalized to other countries in sub-Saharan Africa. Until there are robust and standardized geographic data available for sub-Saharan countries such as Liberia, individuals working in these countries will need to develop their own geospatial datasets. This protocol outlines one method of incorporating local knowledge, GPS data collection, and publicly available geospatial resources to develop a comprehensive geospatial dataset. As more geographic data are collected in these countries, especially in rural areas, and added to open source platforms such as OSM, the challenges associated with geospatial data collection will be reduced and these resources will be more widely available for future work.

## Limitations

The process we described has several limitations worth noting. First, all MWH logbook data was recorded by hand by nurses or midwives at the primary healthcare facility, thus spelling and legibility issues may have contributed to the challenges in village identification. Second, healthcare facilities did not have a master list of villages that fall within their catchment areas. While individuals working at the healthcare facilities were able to provide our team with a list of the larger and more well-known communities in their catchment areas, this list did not include smaller or more distant villages. When prompted, healthcare workers could usually identify the relative location of smaller villages, however, there was no way to verify completeness. While most healthcare facilities had hand-drawn maps of their catchment areas, these maps did not include many of the smaller villages within the catchment areas. Without a master list to compare the handwritten logbook entries, it was difficult to verify data points. Finally, it was common for a village to have more than one name or multiple spellings.

Nonetheless, while the initial list of villages generated from the MWH logbooks required extensive cleaning and processing, our protocol’s step-wise approach and incorporation of a diverse set of resources allowed for the collection of GPS coordinates for the home village of 93.7% of the women who accessed the MWHs during our study period. Moreover, by leveraging local knowledge and verifying our results through the triangulation of hand-drawn maps, OSM, and Google Maps, we were able to ensure high quality data.

## Abbreviations

MWHs: maternity waiting homes
GIS: geographic information systems
GPS: global positioning systems
LMICs: low- and middle- income countries
OSM: Open Street Map

## References

[1] Bongaarts J (2016). WHO, UNICEF, UNFPA, World Bank Group, and United Nations Population Division Trends in maternal mortality: 1990 to 2015 Geneva: World Health Organization, 2015. Popul Dev Rev.

[2] Liberia Institute of Statistics and Geo-Information Services (Monrovia) (2014). Liberia demographic and health survey 2013.

[3] Homes WM. A review of experiences. World Health Organisation, Division of Reproductive Health, Safe Motherhood unit, Maternal and Newborn Health. 1996.

[4] Chandramohan D, Cutts F, Chandra R (1994). Effects of a maternity waiting home on adverse maternal outcomes and the validity of antenatal risk screening. Int J Gynaecol Obstet.

[5] Lori JR, Munro ML, Rominski S, Williams G, Dahn BT, Boyd CJ, Moore JE, Gwenegale W (2013). Maternity waiting homes and traditional midwives in rural Liberia. Int J Gynaecol Obstet.

[6] Poovan P, Kifle F, Kwast BE (1990). A maternity waiting home reduces obstetric catastrophes.

[7] Bolstad P (2005). GIS fundamentals: a first text on geographic information systems.

[8] Beyers N, Gie RP, Zietsman HL, Kunneke M, Hauman J, Talley M, Donald PR (1996). The use of a geographical information system (GIS) to evaluate the distribution of tuberculosis in a highincidence community. S Afr Med J.

[9] Tanser F, Wilkinson D (1999). Spatial implications of the tuberculosis DOTS strategy in rural South Africa: a novel application of geographical information system and global positioning system technologies. Tropical Med Int Health.

[10] Van Rie A, Beyers N, Gie RP, Kunneke M, Zietsman L, Donald PR (1999). Childhood tuberculosis in an urban population in South Africa: burden and risk factor. Arch Dis Child.

[11] Wilkinson D, Tanser F (1999). GIS/GPS to document increased access to community-based treatment for tuberculosis in Africa. Lancet.

[12] Kalipeni E, Zulu L (2008). Using GIS to model and forecast HIV/AIDS rates in Africa, 1986–2010. Prof Geogr.

[13] Tanser F, LeSueur D, Solarsh G, Wilkinson D (2000). HIV heterogeneity and proximity of homestead to roads in rural South Africa: an exploration using a geographical information system. Tropical Med Int Health.

[14] Booman M, Durrheim DN, La Grange K, Martin C, Mabuza AM, Zitha A, Mbokazi FM, Fraser C, Sharp BL (2000). Using a geographical information system to plan a malaria control programme in South Africa. Bull World Health Organ.

[15] Craig MH, Snow RW, le Sueur D (1999). A climate-based distribution model of malaria transmission in sub-Saharan Africa. Parasitol Today.

[16] Omumbo J, Ouma J, Rapuoda B, Craig MH, Le Sueur D, Snow RW (1998). Mapping malaria transmission intensity using geographical information systems (GIS): an example from Kenya. Ann Trop Med Parasitol.

[17] Schellenberg JA, Newell JN, Snow RW, Mung’ala V, Marsh K, Smith PG, Hayes RJ (1998). An analysis of the geographical distribution of severe malaria in children in Kilifi District, Kenya. Int J Epidemiol.

[18] Cooper AK, Coetzee S, Kourie DG. Assessing the quality of repositories of volunteered geographical information. 2012.

[19] OpenStreetMap contributors. Bong County, Liberia. 2018.

[20] Google Maps. Bong County, Liberia. 2018.

[21] Lüge T (2015). GIS support for the MSF Ebola response in Liberia.

